# Droplet dynamics affecting the shape of patterns formed spontaneously by transforming UV-curable emulsions

**DOI:** 10.1038/s41598-024-57851-z

**Published:** 2024-03-26

**Authors:** Yoshimi Inaba, Takayuki Yanagisawa

**Affiliations:** Toppan Technical Research Institute, TOPPAN Holdings Inc., Sugito, Saitama, 345-8508 Japan

**Keywords:** Chemistry, Engineering, Materials science

## Abstract

Forming large pitch and depth patterns spontaneously based on a bottom–up approach is a challenging task but with great industrial value. It is possible to spontaneously form an uneven (concave–convex) patterns with submillimeter-to-millimeter-scale pitches and depths by the direct pattern exposure of a UV-curable oil-in-water (O/W) emulsion liquid film. UV irradiation generates a latent pattern of a cured particle aggregation in the liquid film, and an uneven structure is spontaneously formed during the subsequent drying process. This process does not require any printing and embossing plates or development process. In this report, we presented an example of unevenness formation with a maximum pattern depth of approximately 0.4 mm and a maximum pitch width of 5 mm. The patterns formed by this method have raised edges in the exposed areas and fogging in unexposed areas. The pattern shapes become conspicuous under overexposure conditions, but the formation mechanism has not yet been understood in detail and needs to be investigated. In this study, we focused on the exposure process and clarified the mechanism of pattern formation by analyzing the dynamics of emulsion droplets in the medium by an in situ microscopy observation method. As a result, we found that the fogging was mainly caused by light leakage from the exposed area, and the raised pattern edges were caused by droplets transported from the unexposed area to the exposed area. Furthermore, the convection caused by the heat generated from polymerization is a determining factor affecting all these phenomena. By controlling the pattern shape related to convection utilizing direct projection exposure, we showed an example of eliminating raised pattern edges with a height difference of approximately 0.1 mm. By devising and selecting exposure methods, we can expand the range of design applications such as interior decorative patterns.

## Introduction

The engineering process of the bottom–up approach to produce self-organized patterns is not limited to the fabrication of new materials and devices, and it is also attracting attention owing to its advantages in terms of low energy consumption and environmental impact^1^. There are many previous studies on the spontaneous generation of fine patterns. In particular, such studies are aimed mostly at the microfabrication of, for example, semiconductors and diffractive optical elements. On the other hand, there is almost no research on new ideas or process in the direction of spontaneously forming patterns with large pitches and depths. Surface decorative materials imitating natural objects such as wood, leather, and stone are often used for applications such as interior decor materials. This requires a texture that has sensitive feels such as the shading effect and tactile feel that can be obtained from a large pitches and depths pattern. For application examples requiring high functionality, antenna patterns^2,3^ for electromagnetic metamaterials^4–6^ require large pitches on the submillimeter-to-millimeter-scale. To provide optical functions such as anisotropic reflection, a deep asymmetrical sloped shape is required, and more three-dimensional processing within the plane of the substrate is becoming necessary. Conventionally, the top–down manufacturing process for such industrial products not only requires chemicals and cleaning solutions, but also consumes a large amount of energy. For example, in photolithography, waste fluid and energy consumption increase in proportion to thickness. From this background, it is desired to develop patterning technology that simplifies the process and does not emit waste liquid. A self-organizing bottom–up approach will be the effective in solving this problem.

**Figure 1:**
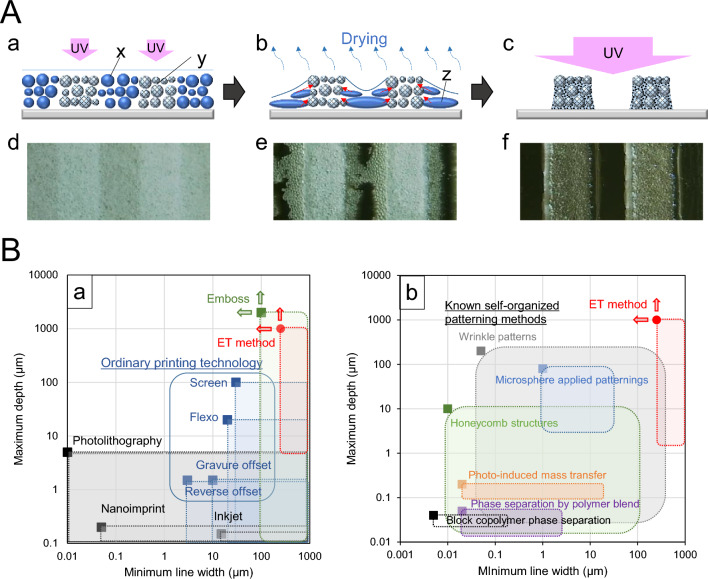
Principle of ET method and its position in patterning technology. (**A**) Schematic of the emulsion transformation method (ET method) for patterning. (**a**) UV exposure of the emulsion liquid film on the substrate to form the pattern. (**b**) Removal of dispersion medium by drying. (**c**) Full UV exposure after the completion of drying. (**d**) Cured particle aggregation pattern formed in liquid film immediately after exposure. (**e**) Spontaneously formed uneven pattern during drying process. (**f**) Completed uneven structure formation. (x) UV-curable emulsion. (y) UV-cured emulsion. (z) Coalesced emulsion droplets. (**B**) Positioning of the ET method. (**a**) Position of the ET method among conventional patterning methods. (**b**) Position of the ET method among known self-organized patterning methods.

In our previous study, we examined the basic principle of the spontaneous concave–convex pattern formation method using a UV-curable emulsion, which we named the emulsion transformation (ET) method^7^. This method is different from the method of manufacturing a top–down-type 3D structure using a high-internal-phase emulsion (HIPE)^8–16^. A crucial difference between these methods is that the ET method does not require a development process to remove unnecessary portions. We used a new approach to the pattern formation by the curing and aggregation of an oil-in-water (O/W) emulsion and then utilized droplet coalescence in the unexposed areas that progresses during the drying of the dispersion medium (Fig. 1A). As drying progresses, droplets of the emulsion liquid coalescing and breaking in the unexposed area, permeate into the voids in the layer of the UV-cured and aggregated particles formed in the exposed area through capillary force, resulting in the spontaneous formation of an uneven pattern. Finally, the entire surface of the layer is irradiated with UV to cure the functional components that have permeated between the UV-cured particles to fix the pattern. This is an original method of forming a pattern by transporting unexposed components horizontally with respect to the substrate surface and entrapping them in an exposed porous layer^7^. We use the expression “spontaneous” was to emphasize that particle aggregation in the medium and the destruction of emulsion droplets and mass transfer proceed naturally toward the exposed area in the drying process, resulting in an uneven structure triggered by UV irradiation. The ET method is a bottom–up patterning induced by UV irradiation. By this method, one can easily obtain large pitches and depths of the submillimeter to millimeter scale.

Figure 1B shows the position of the ET method compared with various representative patterning methods. Among the existing patterning methods by the top–down approach, the ET method can form a deeper pattern than screen printing^17–20^, which corresponds to the area covered by the embossing method^21–23^ (Fig. 1B(a)). On the other hand, Fig. 1B(b) shows the position of the ET method among the self-organized patterning methods. Wrinkle patterns^24–27^ and honeycombs^28,29^ can be formed with relatively large periodic unevenness. In various methods applying microsphere patterning^30–34^, photoembossing^35–38^, and polymer phase separation^39–43^ utilizing polymer blends or block copolymers, large pitches and depths are difficult to form in principle. Therefore, to the best of our knowledge, there is no effective method other than the ET method that easily realizes the pitch and depth of unevenness corresponding to embossing by a self-organizing approach. The ET method can be positioned as a pioneer for unique large-pattern areas among spontaneous structure forming methods.

The ET method is simple and highly efficient for material utilization, has a low environmental impact, and is suitable for industrial applications. The crucial difference between the ET method and other printing and embossing technologies is that it can form patterns with large pitches and depths without a plate. Compared to photolithography, there is no need for a development process. In principle, it is possible to continuously produce patterns using a roll-to-roll process that involves direct projection exposure onto an emulsion liquid film. By switching patterns during continuous web processing, it is possible to manufacture in ultra-small lots at low cost. Since the uneven pattern is formed by coating, exposing, and drying without any development, there is almost no loss of base film due to plate replacement work. The amount of organic waste liquid is also small, so the impact on the environment can be kept to a minimum. To control the characteristics of the obtained uneven structure and make the ET method a practical technology for stable production, it is necessary to study the mechanism of the basic process in detail. In particular, the exposure process is the most important step for controlling the pattern formation. In this study, we investigated the phenomena that occur during exposure and elucidated their effects on pattern formation. Firstly, the photosensitivity curve of the pattern formed by the ET method was obtained along with its appearance. We examined two major features appearing under overexposure conditions: fogging in unexposed areas and the raised pattern edge in exposed areas. Subsequently, the dynamic pattern forming process including aggregation was observed using an in situ microscopic recording system, and the mechanisms of fogging and raised pattern edge formation were elucidated by analyzing recorded video images. The formation of the characteristic shapes was associated with light leakage from the exposed areas and the micro-to-millimeter-scale flow of droplets within the medium. Moreover, in addition to the investigation and control of the pattern shape, we demonstrated the possibility of utilizing the features of more complex patterns themselves for producing decorative designs.

## Results and discussion

### Features of the pattern formed by the ET method

**Figure 2:**
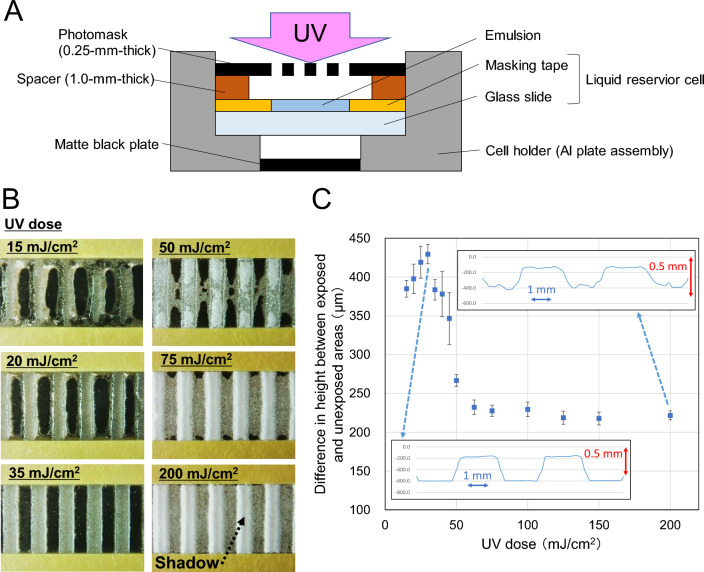
Typical UV dose dependence of patterns obtained by ET method. (**A**) Layout of patterning experiments on UV-curable emulsion. (**B**) UV dose dependence of the stripe pattern appearance. The mean diameter of emulsion particles was 87.7 μm (Fig. S1a). The line/space (L/S) size of the photomask was 2 mm/2 mm. The UV illuminance was 5.0 mW/cm^2^. The patterns were dried at room temperature (23 °C, 48% RH) for 90 min. The patterns were fully exposed to UV at 300 mJ/cm^2^ to fix them. (**C**) Relationship between UV dose and difference in height between UV-exposed and unexposed areas. The pattern heights were determined by measuring the 3D shape and cutting out the cross-sectional profile. Error bars indicate SD. n = 5. Insets show cross-sectional profiles of the patterns exposed to 35 and 200 mJ/cm^2^.

The layout of the patterning is shown in Fig. 2A. A liquid reservoir cell was filled with an emulsion whose composition is shown in Table S1. Thereafter the liquid surface was irradiated with UV light through a photomask. Then, the pattern was spontaneously formed by drying at room temperature. After drying, the entire film surface was irradiated with UV to fix the pattern.

Figure 2B shows the relationship between the UV dose and the stripe pattern appearance as a representative example. Figure 2C indicates the relationship between the UV dose and the pattern height corresponding to Fig. 2B. As shown in Fig. 2B, at low exposure doses of 15 and 20 mJ/cm^2^, the aggregated cured particles are unstable owing to insufficient curing, and the deformation of the pattern and the disordered edges are conspicuous. At 35 mJ/cm^2^, the pattern shape is sharp (Fig. 2B) and the maximum pattern height is obtained (Fig. 2C). When the UV dose is further increased, cured particles start to appear on the unexposed areas from around 50 mJ/cm^2^ (Fig. 2B). At 75 mJ/cm^2^ or higher, the unexposed areas are almost filled with cured particles, giving them the appearance of “fogging” in terms of conventional photographic technology, and the pattern height decreases (Fig. 2C). Moreover, as shown in the photograph of the area exposed to a UV dose of 200 mJ/cm^2^, the edge of the pattern of the exposed area is raised and a shadow is observed at the center. The existence of the raised pattern edge is also supported by the shape of the cross-sectional profile shown in the insets in Fig. 2C. Regarding the features of the pattern shape generated by the ET method, “fogging” is hereinafter referred to as the “ET fog” and the “raised pattern edge” as the “ET edge”.

### Behavior of emulsion in the medium triggered by UV irradiation

**Figure 3:**
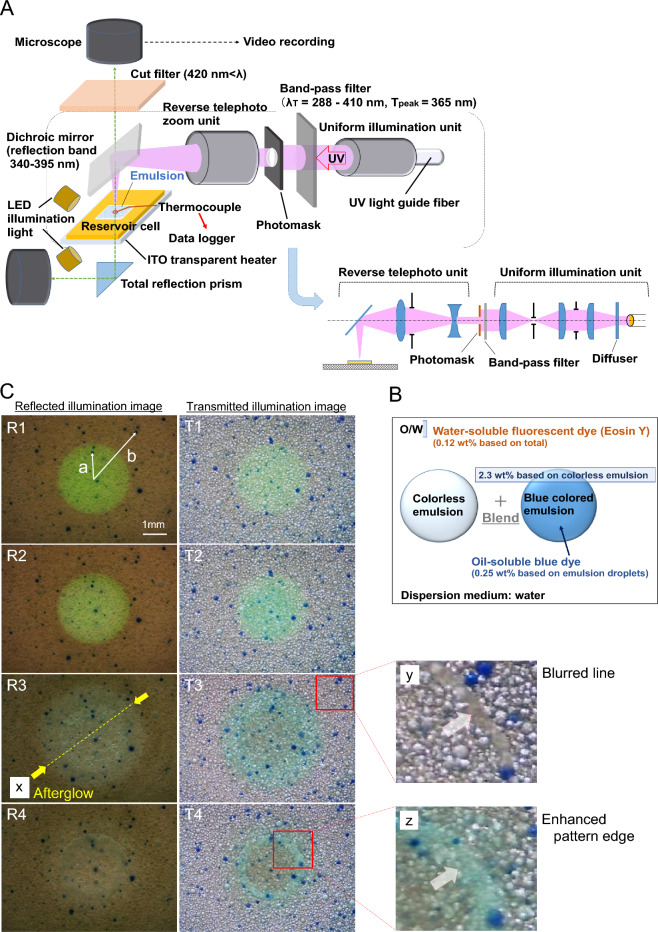
In situ microscopy observation of emulsions in the medium for UV pattern exposure. (**A**) Illustration of in situ observation apparatus (Fig. S2). All lens units were made of synthetic quarts. The UV light guide fiber was connected to a short arc SHP light source. The illuminance of the UV circular pattern was 11.25 mW/cm^2^. (**B**) Illustration of colored emulsion composition for observing emulsion dynamics. (**C**) Cropped video images of the emulsion in UV circular pattern exposure process (Movies S1 and S2). R1 to R4 were taken with reflective illumination, and T1 to T4 were taken with transmitted illumination. R1 and T1 were taken just before aggregation induced by UV exposure occurred. R2 and T2 were taken immediately after the aggregation was completed. R3 and T3 were taken just after the end of UV irradiation. R4 and T4 were taken just before the end of video recording. R1 to R4 were taken at elapsed times of 3.70, 5.20, 15.17, and 71.59 s from the start of UV irradiation at UV exposure doses of 41.6, 58.5, 170.7, and 170.7 mJ/cm^2^, respectively. T1 to T4 were taken at elapsed times of 3.71, 5.18, 15.45, and 76.13 s from the start of UV irradiation at UV exposure doses of 41.7, 58.3, 173.8, and 173.8 mJ/cm^2^, respectively. Arrows (a) and (b) shown in R1 were defined, and the change in distance from the center of the circular exposed area to the inside and outside of the exposed area was measured. (x) Afterglow. (y) Blurred line. (z) Enhanced pattern edge. The mean diameter of emulsion particles was 92.9 μm (Fig. S1b).

The movement of emulsion during exposure was recorded using a system for pattern exposure and observation shown in Fig. 3A (see photo in Fig. S2). This observation system has the same principle of a fluorescence microscope, and it can project patterns and has a long working distance. The illumination system has a Köhler illumination^44,45^ lens arrangement, and the imaging system has a reduction projection lens configuration^46^. A water-soluble fluorescent dye (eosin Y) was added to the emulsion to enable the differentiation between UV-pattern-exposed and -unexposed areas based on the fluorescence color (Table S2). The emulsion containing an oil-soluble blue dye (Kayaset Blue N) in trimethylolpropane triacrylate (TMPTA) was prepared to identify individual emulsion droplets and to easily observe their movement (Table S3). A mixture of approximately 2.3% by weight of the blue emulsion to the emulsion containing eosin Y was used as a specimen (Fig. 3B and Table S4).

Figure 3C shows cropped video images of the emulsion before and after aggregation during and after UV circular pattern exposure. Transmitted illumination (Fig. 3C (T1–T4)) was used in addition to reflected illumination (Fig. 3C (R1–R4)) for observation so as not to overlook any phenomena. In R1 in Fig. 3C, the yellow-green circular portion is the UV-exposed area, which was identified by the eosin Y fluorescence. For the arrow in (a), the motion of the emulsion was measured by determining the change in distance inside the circular pattern and following this change in over time. The arrow in (b) was defined to analyze the movement of the unexposed area. Along the length of these arrows, blue particles were utilized as markers to keep track of their motion. The lengths of the arrows were measured by printing out the cutout image and using vernier calipers. From the images in Fig. 3C, three features can be identified: (x) afterglow, (y) blurred line, and (z) enhanced pattern edge. In particular, the (z) enhanced pattern edge can be identified with the ET edge and inferred to have already been formed during the exposure stage.

The afterglow shown in Fig. 3C (x) will be discussed below. The fluorescence emission area extends outside the exposed area (Movie S1). In the images with transmitted illumination in Fig. 3C (T1–T2), the time required for the exposed regions to aggregate was 1.47 s. At this point, the excited eosin Y fluorescence does not extend outside the exposed region (Movie S2). Figure 3C shows T3 taken after the end of UV irradiation, and the afterglow extends outside the exposed area. Afterglow weakened at 60.68 s after the end of UV irradiation (Fig. 3C (T3–T4)). Since eosin Y was dissolved in the aqueous phase, the spread of afterglow indicates the area reached by the dispersion medium moving from the exposed area.

**Figure 4:**
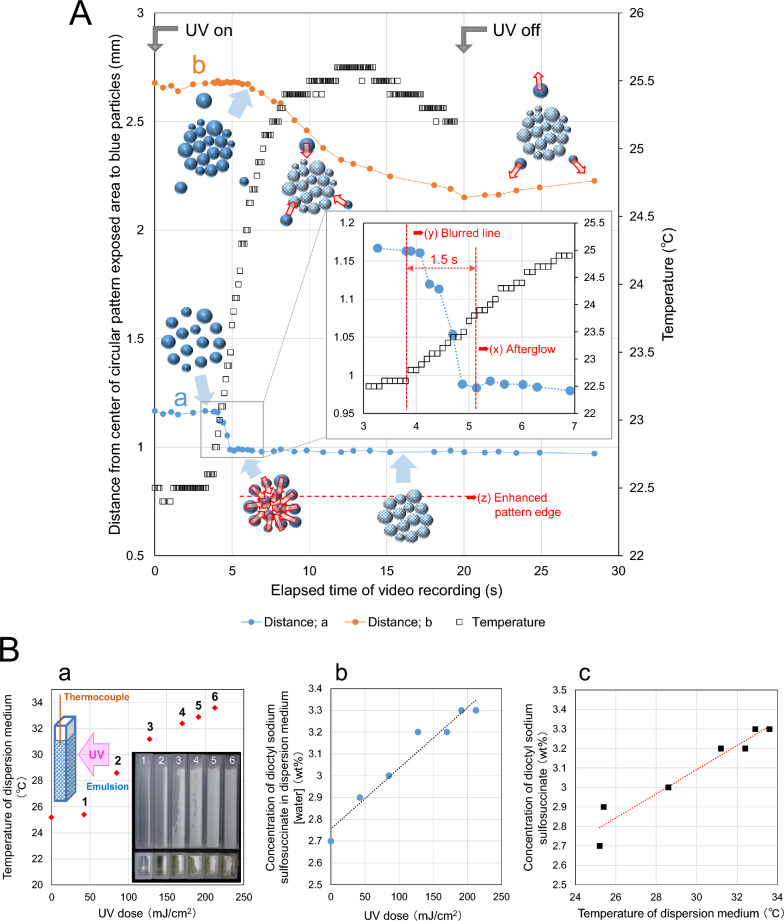
Emulsion dynamics and changes in emulsifier concentration in the medium due to UV irradiation. (**A**) Quantitative analysis of emulsion movements. (Plots a) Aggregation behavior of cured emulsions inside the circular pattern exposed area (Fig. 3C, Movies S1 and S2). (Plots b) Movement of emulsion droplets outside the circular pattern exposed area (Fig. 3C, Movies S1 and S2). Distances in (a) and (b) correspond to the arrows shown in Fig. 3C(R1). (**B**) Changes in free emulsifier concentration in the medium with UV dose. (**a**) Temperature rises during UV irradiation. The inset photo shows the side and top openings of the cell after the removal of unaggregated residues. (**b**) UV dose dependence of free emulsifier (dioctyl sodium sulfosuccinate) concentration in aqueous phase. R^2^ = 0.951. (**c**) Correlation between temperature and the concentration of free emulsifier in water. R^2^ = 0.931. The mean diameter of particles of emulsion employed in this analysis was 82.8 μm (Fig. S1c).

Figure 4A (Plot a) shows the movement of the emulsion over time inside the exposed area as a distance change defined by the arrow in (a). Under the UV illuminance of 11.25 mW/cm^2^, particles moved after an induction period of about 3.7 s from the start of irradiation and almost completely stopped after 5.2 s (Movie S1). Aggregation occurred as the temperature increased and completed in approximately 1.5 s, which reduced the initial distance by approximately 16%.

Figure 4A (Plot b) shows the movement of droplets outside the UV-circular-pattern-exposed area indicated by an arrow in (b) over time. The time at which a droplet in (b) starts moving is about 1.4 s later than in (a). Moreover, the distance (b) decreases until UV irradiation ends and increases after the end of UV irradiation. Since the direction in which the afterglow spreads is opposite to that in which (b) moves, convection in the liquid film is suggested. The surrounding dispersion medium is dragged by the movement of aggregation in the exposed area, and the uncured emulsion droplets move toward the exposed area and converge. As a result, the distance in (b) decreases initially, but when the UV irradiation ends and the temperature of the exposed area begins to drop, the emulsion droplets in the unexposed areas that have been dragged around the exposed area move back to their initial location. This movement indicates that the distance in (b) begins to increase after the exposure ends. The distance in (b) decreases by about 20% from the start to the end of UV irradiation over a period of 20 s. Moreover, the distance in (b) from the end of UV irradiation to the end of video recording increases by about 4% over a period of 8.4 s.

Figure 4B shows the increase in emulsifier concentration in the aqueous phase due to the temperature increase caused by UV irradiation. Details of sample collection methods are given in Materials and Methods (refer to “Quantitative analysis of emulsifier concentration”). The basic emulsion model (Table S1) was used for this analysis. The inset in Fig. 4B (a) shows the cell sides and open top after the immediate removal of the uncured components after exposure. A residual white aggregated layer sticking to the sides of the cells was observed. Figure 4B (b) shows the relationship between the dose and the emulsifier concentration in the aqueous phase, and Fig. 4B (c) shows the relationship between the temperature and the emulsifier concentration. These results indicate that there is a positive correlation between the temperature rise due to exposure and the free emulsifier (dioctyl sodium sulfosuccinate) concentration in the dispersion medium. This correlation can support rapid aggregation mechanisms. This is because the emulsifier desorbed from the droplet surface owing to the temperature rise caused by polymerization, the surface potential dropped rapidly, and the dispersed state became unstable^7^.

The blurred line in Fig. 3C (y) spreads outside the exposed area immediately after irradiation and narrows after irradiation (Movies S1 and S2). This spread of the blurred line is faster than the afterglow spread and appears to be related to surface flows^47^. It is presumed that the emulsifier desorbed from the cured particles was concentrated at the air interfaces and move to the unexposed areas along the edges of the surface flow region generated by the temperature gradient. We speculate that the portion appearing to be a blurry line corresponds to the area with locally high emulsifier concentration and has a different refractive index than the surrounding area.

### Causes of ET fog in unexposed areas

**Figure 5:**
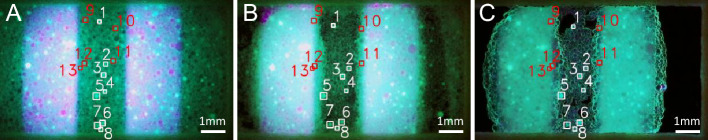
Light leakage to the unexposed area. (**A**) Immediately after UV exposure. (**B**) Just before the end of UV exposure (19.52 s elapsed). (**C**) After drying (166.72 s elapsed). (1) to (13) marked with squares are coumarin-6-containing emulsions that emit fluorescence owing to light leakage from exposed to unexposed areas (Movie S3). The UV illuminance was 85.3 mW/cm^2^ and the exposure time was 20 s. The coumarin-6-containing emulsions have a mean diameter of 88.9 μm (Fig. S1g). Cropped video images were taken from the back side.

To investigate the cause of ET fog, an emulsion containing a fluorescent dye (coumarin 6) was prepared (Table S5). The coumarin-6-containing emulsion was blended with the uncolored emulsion at 5 wt% on the basis of the total amount of the emulsion composition (Table S6). The coumarin-6-containing emulsion was exposed to a UV stripe pattern for about 20 s, and immediately after the exposure was finished, it was dried by heating at 80 °C with an indium tin oxide (ITO) heater placed under the liquid reservoir cell. Figure 5 shows cropped video images of coumarin 6 particles emitting fluorescence when UV light leaks into the unexposed area. Particles marked (1)–(13) in unexposed areas during pattern exposure show fluorescence emission similarly to those in exposed areas; this was because they were exposed to UV light leakage. Particles (1)–(8) clearly show fluorescence under UV illumination after drying. It was found that ET fog is formed by the diffusion of UV light leakage because the positional relationship of fluorescent particles during exposure does not change markedly even after drying (Movie S3).

To quantitatively understand the relationship between ET fog formation and bulk flow, we again used the blue emulsion as a marker. The blue emulsion was blended with the uncolored emulsion at 20 wt% based on the total amount of the emulsion composition (Table S7). The liquid film was exposed to UV for 20 s and dried by heating at 80 °C, similarly to the case of using the fluorescent dye described above. Videos were taken from both the back and front sides (Movies S4 and S5).

**Figure 6:**
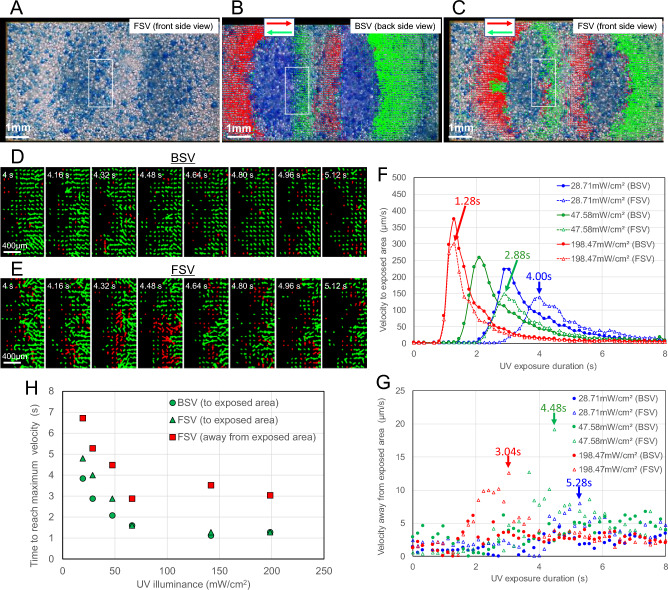
Analysis of the flow generated in the boundary area between the unexposed and exposed areas. (**A**) Defining the region of interest (ROI) indicated by a white rectangle. The mean diameter of blue emulsion particles was 77.5 μm (Fig. S1h). (**B**) Vectors indicating the flow velocity distribution from the back side view (BSV) (Movie S4). Red vectors indicate ± 90° in the positive direction of the x-axis, and green vectors show ± 90° in the negative direction. The length of a vector is the amount of movement per unit time, expressed as 5 times the pixel size/0.16 s. 4.48 s elapsed from the start of UV irradiation. The UV illuminance was 47.58 mW/cm^2^. (**C**) Vectors indicating the flow velocity distribution from the front side view (FSV) (Movie S5). The definitions of vectors are the same as those in (**B**). The elapsed time from the start of UV irradiation and the UV illuminance are the same as those in (**B**). (**D**, **E**) Enlarged time-series diagram of the ROI vector. (**F**) Correlation between UV exposure duration and velocity of flow to exposed area. (**G**) Correlation between UV exposure duration and velocity of flow away from exposed area. (**H**) Correlation between UV illuminance and time to reach maximum velocity.

Figure 6 shows the flow analysis results of droplets in the medium during exposure. The video images were cropped 4.48 s after the start of exposure (Fig. 6A–C). When analyzing the video images, we defined a region of interest (ROI) indicated by a white rectangular frame at the boundary between the exposed and unexposed areas (Fig. 6A). Figure 6B and C are images of the bottom and front surfaces of the droplet layer, respectively, with the velocity vectors of the particles added. The red velocity vector indicates the flow toward the right of the figure, and the green velocity vector indicates the flow in the opposite direction (toward the left of the figure) in the ROI. Figure 6D and E show the vector distribution in time series at around 4.48 s from the start of exposure. From the back side view (BSV), droplets moving from the unexposed area to the exposed area predominate, as shown in the green velocity vector diagram (Fig. 6D). On the other hand, from the front side view (FSV), there are many droplets moving from the exposed area to the unexposed area as shown in the red velocity vector diagram at an elapsed time of around 4.48 s (Fig. 6E). On the surface of the droplet layer, flows of red velocity vectors were mixed with opposite flows of green velocity vectors, resulting in confusion (Fig. 6E).

To quantitatively clarify these flows, we investigated the relationship between the UV exposure duration and the velocity of droplets (Fig. 6F and G). From Fig. 6F, the movement of a droplet observed from the FSV starts slower than that from the BSV. At a higher UV illuminance, the difference in the time of droplet movement becomes smaller between the FSV and the BSV, and the induction period becomes shorter. On the other hand, the flow away from the exposed area is shown in Fig. 6G. Although there are variations in the data, the flow velocity on the FSV tends to be higher. In addition, the maximum velocity of flow away from the exposed area is lower than that of the flow to the exposed area (Fig. 6G), as compared with the results in Fig. 6F. This decrease in velocity suggests the existence of convection rising from the bottom of the unexposed area to the surface of the liquid film along the boundary wall of the exposed areas.

Figure 6H shows the relationship between the UV illuminance and the time to reach the maximum velocity. Focusing on the UV illuminance of 100 mW/cm^2^ or lower, the time to reach the maximum velocity is about 1 s longer for the surface flow (FSV, see green circles) than for the bottom flow toward the exposed area (BSV, see green triangles). Furthermore, the time to reach the maximum velocity of the movement away from the exposed area (FSV, see red squares) is 1–2 s longer than that of the movement from the surface of the droplet layer to the exposed area (FSV, shown in green triangles). This supports the existence of natural convection caused by heat transfer from the exposed area to the unexposed area. The upward flow velocity in the unexposed area should be maximum near the boundary wall of the exposed area. The droplets at the bottom of the liquid film begin to move as they are dragged by the upward flow along the boundary, and the momentum is transferred to the droplets on the surface layer with a slight delay. When the upward flow along the boundary wall reaches the interface with air, droplets begin to move toward the unexposed area. In addition, owing to light leakage, the number of cured and aggregated droplets that stopped moving at the boundary wall of the exposed areas increases.

**Figure 7:**
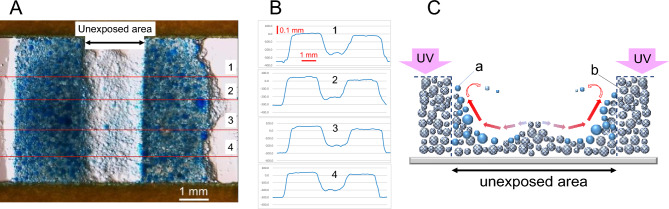
Cross-sectional shape of ET fog layer and its formation mechanism. (**A**) Transmitted illumination micrograph of a pattern with ET fog (Movie S6). A striped UV pattern with an illuminance of 113.6 mW/cm^2^ was applied for about 20 s. (**B**) Cross-sectional profile of ET pattern. (1)–(4) correspond to the cutting positions in photo (**A**). The middle portion of the profile represents the unexposed area, which corresponds to the position where ET fog occurred. (**C**) Illustration of the formation mechanism of the raised shape formed in the middle of ET fog. Arrows qualitatively represent the direction and magnitude of heat flow. (x) Crude emulsion droplets. (y) Cured droplets.

The raised portion was observed at the center of the unexposed area after drying (Fig. 7A and B). The composition of the emulsion mixture used for the observation is shown in Table S7. The movement of the central portion is small (Movie S6). This is because the heat generated in the exposed portions on both sides induces convection in opposite directions to the left and right, and the forces are canceled in the central portion; thus, the particles tend to stay in place (Fig. 7C). Owing to the light leakage, the curing of the emulsion in the unexposed area progresses, but if the flow of the bottom toward the exposed area is slow, the cured and aggregated emulsion particles tend to remain in their initial positions. Moreover, if the flow at the bottom is fast, particles are dragged by convection and accumulate at the edge wall of the exposed portion at a higher proportion than those remaining in their initial positions, resulting in their aggregation owing to the higher level of light leakage. In general, even with a photoresist, it is often observed that pattern height differences decrease owing to overexposure, as in ET fog. The main reason why layers remain in the unexposed areas after overexposure is often light leakage^48,49^. A major cause of ET fog is also light leakage to the unexposed areas. In the case of the ET method, it is also necessary to consider the contribution of cured particles moved by convection. However, it is difficult at present to clarify the extent of their involvement in fogging; this is an issue that will be addressed in our future studies.

### Mechanism of ET edge generation

**Figure 8:**
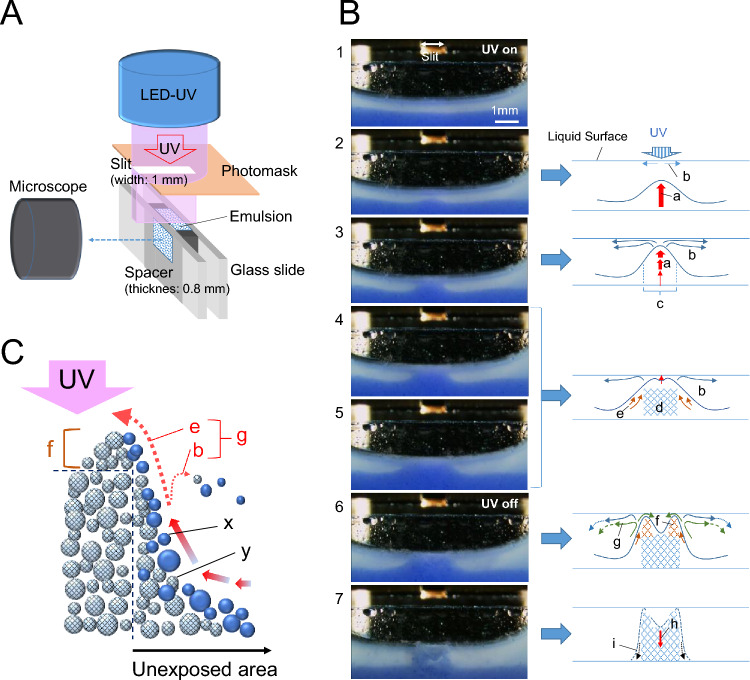
In situ observation of emulsion liquid film from the cross-sectional direction. (**A**) Illustration of cross-sectional observation setup (Fig. S3). The spacing between glass slides was set to 0.8 mm. A photomask with a thickness of 0.25 mm and a 1-mm-wide slit was placed 1 mm above the cell. Exposure was performed using a UV-LED light source (λ = 365 nm; illuminance, 9.8 mW/cm^2^). (**B**) Cut-out images from the cross-sectional direction of the emulsion liquid film and illustrations of the flow phenomenon (Movie S7). A 10 wt% of the basic emulsion model (Table S1) was colored with a blue dye (Kayaset Blue A-2R) for easy observation. A 0.2 wt% blue dye was incorporated in TMPTA droplets. The mean diameter of emulsion particles was 14.9 μm (Fig. S1f). A cell filled with emulsion was left for 7 min to form a supernatant layer for the easy observation of the movements. (1) Start of UV slit exposure. (2–7) show elapsed times of 3, 4, 6, 17, 57, and 177 s, respectively. The total exposure time was 59 s at 578.2 mJ/cm^2^. (a) Ascending flow due to buoyancy. (b) Surface-tension-driven flow. (c) Polymerization and aggregation progression region. (d) Fixed primary aggregation region. (e) Natural convection. (f) Fixed secondary aggregation region. (g) Confluence flow (b + e). (h) Subduction caused by temperature drop. (**C**) Illustration of ET-edge generation mechanism. (x) Crude emulsion droplet. (y) Cured emulsion. (b), (e), (g), and (f) are the same as in (B).

We attempted to clarify the ET edge formation mechanism by observing the emulsion layer from the cross-sectional direction. The observation setup shown in Fig. 8A (see the photo in Fig. S3) was assembled, and the movement of the emulsion generated during partial UV irradiation was recorded from the cross-sectional direction of the liquid film. Figure 8B shows time-series images cut out at specific elapsed times from the video and a schematic illustration showing the motion of the emulsion corresponding to each image (Movie S7). The pattern of emulsion movements in the medium is explained below on the basis of the illustration in Fig. 8B, and the mechanisms will be discussed.

At a UV dose of 29.4 mJ/cm^2^ (Fig. 8B (2); 3 s after UV irradiation), two types of flow were observed. One is the upward flow (a) due to the buoyancy induced by the heat generated by polymerization. The other is the flow (b) probably driven by the surface tension gradient (Marangoni effect)^47^. A partial temperature rise of the liquid surface causes a difference in surface tension between the exposed and unexposed areas.

When the dose is from 39.2 to 58.8 mJ/cm^2^ (Fig. 8B (2, 3); 4 s to 6 s elapsed), the upward flow continues owing to buoyancy. A surface-tension-driven flow (b) is generated toward the unexposed area at the time when the boundary surface of emulsion sedimentation raised by the effect of the upward flow (a) reaches the liquid surface. It propagates along the liquid surface. During this process, the curing and aggregation of droplets proceed simultaneously in the exposed area defined by the polymerization and aggregation progression regions (c).

After the exposure at 68.6 mJ/cm^2^ (Fig. 8B (4, 5); for 7 s), the aggregation of the polymerized particles in the exposed area (c) is completed and the fixed primary aggregation region (d) stops rising. At that time, the movement of the exposed portion stops owing to aggregation. However, the heat generated by polymerization is transported to the boundary between the exposed and unexposed areas. Therefore, natural convection (e) toward the liquid surface continues in the uncured emulsion droplets present in the periphery of the region (d).

At doses higher than 78.4 mJ/cm^2^ (Fig. 8B (5, 6); over 8 s), uncured droplets reach the liquid surface through natural convection (e). By partially covering the edges of the UV-exposed area with the rising flow (g), the fixed secondary aggregation region (f) is newly formed in the upper peripheral portion of the region (d). Moreover, the flow (b) to the unexposed area also continues to occur. At this stage, some of the emulsion droplets move to the exposed area by convection (g), which is a combination of flows (e) and (b). Therefore, it can be concluded that the ET edge is a fixed secondary aggregation region (f) formed mainly by the flow (g) consisting of flows (e) and (b) (Fig. 8C). The emulsion carried by the flow (b) to the unexposed areas is presumed to be part of the cured emulsion near the exposed areas and uncured droplets.

UV irradiation was stopped after 59 s and remained turned off for 115 s (Fig. 8B (6, 7)). The total UV dose was 578.2 mJ/cm^2^. When the irradiation is stopped, the heat generation due to polymerization stops and the temperature decreases. As a result, the polymerized, aggregated, and fixed particles, which have floated owing to the buoyancy caused by the temperature rise, cool and sink (h). At the same time, sedimentation (i) also occurs in the uncured emulsion droplets that had continued to rise around the periphery of the cured region.

### Control of pattern shape and utilization of features

**Figure 9:**
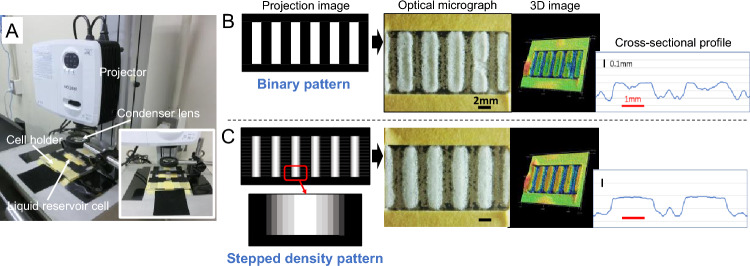
Pattern edge shape control utilizing projection direct exposure. (**A**) Projection exposure setup. The condenser lens (biconvex lens; diameter, 50 mm) made of BK7 glass has a focal length of 100 mm. The mean diameter of particles of the emulsion used was 96.8 μm (Fig. S1d). (**B**) Formation of a binary stripe pattern. The duration of exposure was 10 s at a visible light intensity of 0.571 W/cm^2^. The pattern-exposed emulsion was dried at room temperature (24 °C 37%RH) for 90 min. After drying, the formed pattern was fixed by full UV exposure at 300 mJ/cm^2^ with an illuminance of 5 mW/cm^2^ using an LED-UV (λ = 365 nm) light source. (**C**) Formation of a stepped density stripe pattern. Exposure, drying, and fixation conditions are the same as in (**B**). 3D images were stretched three times in the depth direction to emphasize the stereoscopic effect.

A commercially available visible range digital light processing (DLP) projector with a high-pressure mercury lamp was employed as a light source. The spectrum of the projector is shown in Fig. S4. Figure 9A (see the setup in Fig. S5A) shows the layout of the projection exposure experiment. A condenser lens was installed in front of the projected image to ensure the required light intensity, and maskless pattern irradiation was performed. To effectively utilize the short wavelength band of the visible region, bis(2,4,6-trimethylbenzoyl) phenylphosphine oxide (BAPO) was employed as a photoinitiator in the emulsion to ensure sensitivity (Table S8).

Figure 9 shows the shape change of the ET edge caused by the projection exposure of the density modulation pattern. Figure 9B shows the result of forming a binary stripe pattern with sharp edges. Micrographs, 3D images, and cross-sectional profiles clearly show the ET edges formed. Chip defects seen in parts of the pattern were caused by the bursting of air bubbles in the emulsion liquid as the liquid was injected into the cell. On the other hand, as shown in Fig. 9C, the ET edge can be eliminated the height difference of approximately 0.1 mm and the shape of the pattern edge can be controlled by applying a density gradient to the pattern edge. This approach is to locally reduce the temperature rise due to rapid polymerization and suppress the convection in the liquid film. The method reduces the amount at which UV-unexposed droplets reach the top areas of the aggregating patterns owing to convection and suppresses the formation of fixed secondary aggregation regions.

**Figure 10:**
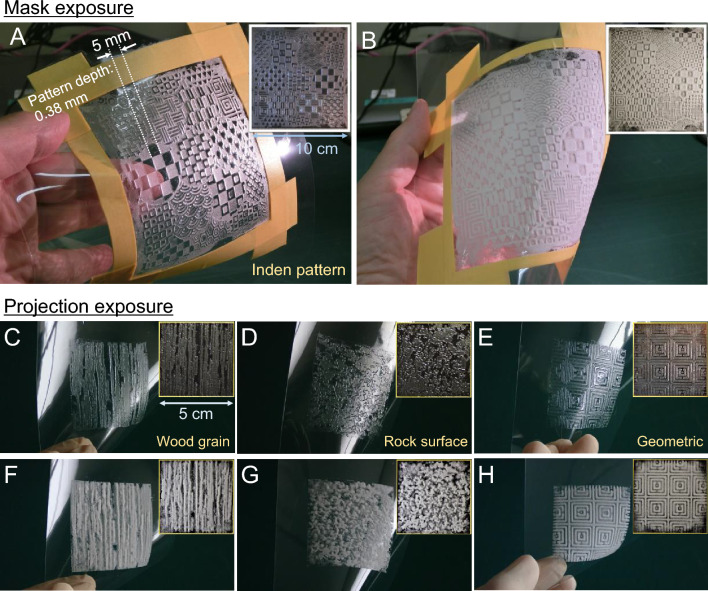
Examples of patterns formed on plastic film. (**A**) Pattern formed with appropriate exposure dose providing maximum depth. The pattern size was 10 cm × 10 cm. The patterning layout used for mask exposure is shown in Fig. S5B.. A 75-μm-thick polyethyleneterephthalate (PET) film was used as the substrate. The mean diameter of emulsion particles was 89.3 μm (Fig. S1e). The photomask was prepared by fixing a black toner pattern on a 100-μm-thick PET film with a laser printer. The distance between the photomask and the emulsion liquid surface was approximately 2.5 mm. Exposure at 29 mJ/cm^2^ with an illuminance of 5 mW/cm^2^ using LED-UV (λ = 365 nm) light source. Subsequently, drying was carried out at room temperature (23 °C, 48% RH) for 150 min. After drying, the surface was fixed by full UV exposure at 300 mJ/cm^2^. (**B**) Pattern formed with overexposure. Exposure at 150 mJ/cm^2^ with an illuminance of 5 mW/cm^2^. Other experimental conditions, the size of the pattern, and the equipment, materials, and procedures used were the same as those in (**A**). (**C**–**H**) Various examples of patterning by projection exposure. The patterning layout used for projection exposure is shown in Fig. S5C. The pattern dimensions were 5 cm × 5 cm. The emulsion used has a mean particle size of 96.8 μm (Fig. S1d). A 75-μm-thick PET film was used as the substrate. The projected image size was reduced by a condenser lens (biconvex lens; diameter, 100 mm) made of BK7 glass with a focal length of 300 mm. The visible projection light intensity at the exposed surface was 89.5 mW/cm^2^. (**C**–**E**) Appropriate exposure duration: 10 s. (**F**–**H**) Overexposure duration: 60 s. (**C**–**H**) The masking tape for the liquid reservoir cell was removed. All patterns obtained by projection exposure were dried at room temperature (23 °C, 41% RH) for 100 min. After drying, the patterns were fixed by full UV exposure at 300 mJ/cm^2^ with an illuminance of 5 mW/cm^2^ using an LED-UV light source.

In principle, the main cause of ET fog is light leakage; therefore, it is necessary to select appropriate exposure conditions to suppress it. In Fig. 10, the composition of emulsions used for mask and projection exposures shown in Tables S1 and S8, respectively. Figure 10A, C, D and E show examples of patterns fabricated with an appropriate UV dose on a 75-μm-thick polyethyleneterephthalate (PET) film substrate. Figure 10B, F, G and H show examples of the same pattern prepared with overexposure. From these translucent and snow-like appearances, one supposes that both can be useful for providing texture in decorative design applications. We expect that the overexposed white snow-like appearance can be utilized in interior decorative design. In addition to decorative design applications, the ET method is also expected to be applicable to products frequently used in embossing technology, e.g., light diffusion^50–52^; the fabrication of anisotropic light reflection^53^ films and sheets, and microfluidics such as microwells^54–57^ and microchannel^58,59^ parts; and braille^60,61^ formation.

## Conclusion

Focusing on the exposure process of patterning by the ET method, we extracted the main phenomena that provide the characteristics of the formed patterns and analyzed their mechanisms. From the results, we demonstrated the control of the pattern shape and the utilization of the features themselves by projection exposure. Under overexposure conditions, the resulting pattern has the ET edge, and the unexposed areas form a residual layer of cured particles (ET fog). The predominant factors that generate these phenomena are largely related to the aggregation of cured particles, which is initiated by the temperature rise triggered by photopolymerization. Micro- to millimeter-scale flows such as natural convection involving the surface-tension-driven flow in the medium caused by the heat generated by polymerization have a marked effect. These are the essential photosensitive characteristics in the ET method.

The generation of ET fog is mainly related to light diffusion (light leakage) from the exposed to unexposed areas and to the droplets in the unexposed area carried by convection toward the boundary of the exposed area in the medium. Furthermore, it was suggested that the movement of cured particles should also be considered. The formation of the ET edge is caused by the convection of the droplets carried from the unexposed area to the surface layer of the liquid film along the interface with the cured particle aggregation layer formed by UV irradiation. This surface layer is exposed to UV light, and aggregates at the pattern edge form a raised edge shape.

From this investigation, we found that it is important to create an appropriate density gradient at the edge of the exposure pattern to control the ET edge and select an appropriate dose to control ET fog. For the ET edge, we showed an example in which the edge with a height difference of approximately 0.1 mm was eliminated applying the density gradient exposure. In addition, by applying the ET method to decorative design products, it is possible to intentionally generate ET edges and ET fog to emphasize the appearance and texture of patterns such as shading effects.

It was shown that a pattern with a maximum depth of about 0.4 mm and a maximum pitch width of 5 mm was obtained from the unevenness formation examples related to this verification.

As our future work, we will investigate the effects of solid content, particle size, the specific gravity of droplets, the viscosity of emulsions, and wettability of the substrate, which affect the material transport in the liquid film, on pattern formation. We will also explore the limits of fine patterns and aspect ratios. In addition to these basic studies, we will promote the generation of more practical and expansive decorative patterns.

## Materials and methods

### Materials

Trimethylolpropane triacrylate (TMPTA) (Light Acrylate TMP-A, Kyoeisha Chemical Co., Ltd.) was employed as the oligomer without additional treatment. 1-Hydroxycyclohexyl phenyl ketone (Lunacure 200, DKSH Japan) and bis(2,4,6-trimethylbenzoyl) phenylphosphine oxide (Omnirad 819, IGM Resins B.V.) were used as photoinitiators. An emulsifier (Sanmorin OT-70, Sanyo Chemical Industries, Ltd.) consisting of a mixture of 70% sodium dioctyl sulfosuccinate, 16% propane-1,2-diol (propylene glycol), and 14% water was used as received. Distilled water was used as the dispersion medium. Kayaset Blue N (Nippon Kayaku Co., Ltd.) and Kayaset Blue A-2R (Nippon Kayaku Co., Ltd.) were used as the blue dyes for the oligomer. Eosin Y (Fujifilm Wako Pure Chemical Corp.) was used as the red fluorescent dye for the dispersion medium (aqueous phase). Coumarin 6 (Tokyo Chemical Industry Co., Ltd.) was used as the yellow-green fluorescent dye for the oil phase (TMPTA).

### Emulsification

Into a 50 mL brown vial, the photoinitiator, emulsifier, and oligomer were put one at a time in this order. The vial was rotated for 2 h to mix these materials, and then distilled water was added. Since Omnirad 819 has a slightly low affinity for the oligomer TMPTA, it was dissolved by stirring on a hot plate at 50 °C for 1 h. Eosin Y was added to the emulsified liquid and dissolved by roll stirring for 10 min. Coumarin 6 was previously dissolved in TMPTA at 60 °C for 1 h with appropriate shaking using a natural convection oven. Two emulsification methods were implemented: one using a paint shaker and manual shaking. Emulsification using a paint shaker (PC1171, Asada Iron Works) was carried out for 30 s. The manual shaking involved 10 repetitions of intermittent shaking at intervals of 20 s. The rest intervals provided the time required for the emulsifier as the stabilizer to diffuse to the newly generated interface^62^. After the emulsifying, the vial preparation was further subjected to rotary stirring for 20 min. Tables S1–S8 show the composition of the emulsion model. The size distribution and weight-average particle size of emulsion droplets are shown in Fig. S1. From the perspective of ensuring UV transparency in thick films, we prepared and used an emulsion with large particle diameters (10–100 μm) that are relatively less affected by light scattering.

### Special preparation

A 1.0-mm-thick glass slide was used as a substrate for forming an emulsion liquid film (see Figs. 2A and S5A). Five layers of 80-μm-thick 3 M masking tape were attached to the glass slide. Then, the central part was cut out in the shape of rectangles of 5 mm × 10 mm, 10 mm × 10 mm, 10 mm × 20 mm, and 10 mm × 30 mm to form a bank. This cut-out portion serves as a liquid reservoir cell. A liquid film simulating the coating layer was formed by dropping the emulsion into the liquid reservoir cell, so that the thickness calculated using the specific gravity of the emulsion would be 375 μm. A 1.0-mm-thick aluminum plate spacer was placed on the liquid reservoir cell surface, and a 0.25-mm-thick copper plate used as a photomask was placed on the spacer.

When a PET film was used as a substrate, banks of 100 mm × 100 mm and 50 mm × 50 mm were formed by laying out the masking tape on the glass plate. A liquid reservoir was formed by peeling this embankment from the glass plate and pasting it on the PET film. The PET film with a liquid reservoir was fixed on a suction plate, and a nitrile rubber spacer with a thickness of 2 mm was placed thereon, and the emulsion was injected into the embankment. After that, a pattern was printed on the 100-μm-thick PET film using a laser printer and placed on the spacer as a photomask (Fig. S5B). The photomask was removed from the liquid reservoir after completing the UV exposure of the pattern, and the cell was dried. After drying, the entire surface was exposed to UV for fixing.

Three types of light source were employed. LED-UV (H-1VH4-V1 equipped with the uniform-illumination lens unit HDL-Q1, Hoya Candeo Optronics) was used in laboratory patterning experiments. A fiber light guide UV source (LS-165UV, DC 165 W short arc SHP lamp, Sumita Optical Glass, Inc.) was used in the in situ observation experiment. A digital light processing (DLP) projector (PJ WX2440, 190 W high-pressure mercury lamp, Ricoh Company, Ltd.) was used in maskless direct patterning experiments.

Two methods were employed for drying: high-temperature (80 °C) and room-temperature drying. In the in situ observation experiment, the ITO transparent heater (glass heater unit SS-052, Blast Co., Ltd.) was placed directly under the liquid reservoir cell, and heating was started immediately after UV irradiation ended. The temperature reached 80 °C in about 20 s.

### Observation and measurement

To obtain the size distribution of emulsion droplets, the weight-average diameter was measured using a particle size distribution measuring system (Microtrac MT3300EXII, Nikkiso Co., Ltd.) equipped with a liquid circulation pump (Microtrac USVR, Nikkiso Co., Ltd.). For video and still-image shooting, a zoom microscope (L-815, Hozan Tool Ind. Co., Ltd.) equipped with a USB camera (L-835, Hozan Tool Ind. Co., Ltd.) was used. The height difference of the pattern was obtained by measuring the shape of the uneven pattern and extracting the cross-sectional profile. Aluminum with a thickness of 800 Å was formed on the surface of the patterned specimen using vacuum vapor deposition equipment (VC-500P, JEOL Ltd.). Then, a three-dimensional (3D) shape was measured using a one-shot 3D shape measuring instrument (VR3100, Keyence Corp.). The height difference of the pattern was directly obtained from the cross-sectional profile data. A film thickness gauge (TH-104, Tester Sangyo Co., Ltd.) was also used to determine the pattern depth and thickness of the film substrate.

The UV exposure dose was measured using a photometer (ORC Light Measure Model UV-351, ORC Manufacturing Co., Ltd.). The spectrum of the projector was measured using an optical fiber spectrometer (FLAME-S-XR1, Grating_#XR1—500 line/mm blazed at 250 nm, P400-1-SR 400 μm optical fiber 1 m, Ocean Optics, Inc.). The total dose of visible light generated by the projector was measured using a power meter (StarBright, combined with high-sensitivity thermal sensor Model 3A, Ophir Optronics Solutions Ltd.).

### Quantitative analysis of emulsifier concentration

The emulsion liquid temperature was measured using a thermocouple (TH-8181-1-M, ThreeHigh Co.,Ltd. Japan, JIS standards Class 2) while UV irradiation was applied to the emulsion in the two-sided transparent glass cell. The measured temperature at the end of UV irradiation was used as the liquid temperature. The illuminance of the LED-UV light source was 10 mW/cm^2^ on the surface of the glass cell. The illuminance after passing through one surface of the transparent side of the glass cell was 8.5 mW/cm^2^. The exposure dose was varied in the range of 0–212.5 mJ/cm^2^ for six irradiation durations. After the end of exposure, unaggregated residual components were collected from the glass cell into a vial and allowed to stand for 48 h. The supernatant of the leftover liquid was collected, and the emulsifier (sodium dioctylsulfosuccinate) concentration therein was quantified as % by weight. The free emulsifier concentration in the dispersion medium (water) was quantified by ultrahigh-performance liquid chromatography/mass spectrometry (UHPLC/MS) (1260 LC System/6130B Single Quad MS System, Agilent Technologies, Inc.) using a C18 column (ACQUITY UPLC, Waters Corp.).

Note that the unaggregated dispersion components were rapidly collected within 10 s after the end of UV exposure. It was important not to allow the emulsifier, once desorbed from the emulsion surface, to be re-adsorbed to the particles after curing by UV irradiation. In addition, we assumed that the non-uniform distribution of emulsifier concentration would occur between cured and uncured areas owing to UV irradiation, and it was important to sample at an early time before the non-uniform emulsifier concentration returned to its original uniform distribution. Care was taken to complete the sampling while the emulsifier concentration gradient was still present in the dispersion.

### Video image analysis

We used *OpenCV 4.0* as the image processing library. The *TrackerBoosting* function was used to mark and track the coumarin-6-containing fluorescent droplets shown in Fig. 5, and particles 1 to 13 were surrounded by a boundary box in the image immediately after the exposure started. Then, the particles were tracked in a series of still images in a time series of 6.25 fps.

In Fig. 6B and C (flow vector distribution) and Fig. 6D and E (change in flow velocity over time), the flow vector was determined by the optical flow method using the *calcOpticalFlowFarneback* function at 6.25 fps. Flow vectors were obtained at 10 pixel intervals both vertically and horizontally. The arrows in Fig. 6B–E indicate vectors in the range of ± 90° in the right direction shown in red and vectors in the range of ± 90° in the left direction shown in green. The amount of movement at 6.25 fps is indicated by multiplying the movement by 5. The flow velocities in Fig. 6F–H was obtained from the quotient of the movement amount and the period of 6.25 fps. The parameters of *calcOpticalFlowFarneback* have the following values: *pyr_scale* = *0.5*, *levels* = *5*, *winsize* = *11*, *iterations* = *12*, *poly*_*n* = *5*, *poly_sigma* = 5, and *flags* = *0.*

### Supplementary Information

Supplementary Video 1.

Supplementary Video 2.

Supplementary Video 3.

Supplementary Video 4.

Supplementary Video 5.

Supplementary Video 6.

Supplementary Video 7.

Supplementary Information 1.

## Data Availability

The datasets used and/or analyzed during the current study is available from the corresponding author on reasonable request.
