# Supplementary material for: Droplet dynamics affecting the shape of patterns formed spontaneously by transforming UV-curable emulsions
Source: Sci Rep. 2024 Mar 26;14:7102. doi: 10.1038/s41598-024-57851-z (PMC10965956; doi:10.1038/s41598-024-57851-z)
Supplement: Supplementary file 8 — Supplementary Information 1. [file 41598_2024_57851_MOESM8_ESM.pdf]

Supplementary Information

Droplet dynamics affecting the shape of patterns formed spontaneously by transforming of UV-curable emulsions

Yoshimi Inaba[1,*], Takayuki Yanagisawa[1]

[1]Toppan Technical Research Institute, TOPPAN Holdings Inc.; Sugito, Saitama 345-8508 Japan.

Correspondence to: yoshimi.inaba@toppan.co.jp

This PDF file includes the following:

Tables S1 to S8

Figures S1 to S5

Caption for Movies S1 to S7

The other Supplementary Information for this manuscript includes the following:

Movies S1 to S7

Table S1. Composition of basic emulsion model.

Components	Amount (g)
Trimethylolpropane triacrylate	7.5
1-Hydroxycyclohexyl phenyl ketone	0.375
Sanmorin OT-70[(a)]	0.259
Distilled water	9.0

[(a)] Sanmorin OT-70: mixture of 70 wt% dioctyl sodium sulfosuccinate, 16 wt% propylene glycol, and 14 wt% water.

Table S2. Composition of eosin-Y-containing emulsion in the aqueous phase.

Components	Amount (g)
Trimethylolpropane triacrylate	7.5
1-Hydroxycyclohexyl phenyl ketone	0.375
Sanmorin OT-70[(a)]	0.259
Distilled water	9.0
Eosin Y[(b)]	0.02

[(a)] Sanmorin OT-70: mixture of 70 wt% dioctyl sodium sulfosuccinate, 16 wt% propylene glycol, and 14 wt% water.

[(b)]Eosin Y (C.I. Acid Red 87): water-soluble red fluorescent dye.

Table S3. Composition of Kayaset-Blue-N-containing emulsion in the oil phase.

Components	Amount (g)
Trimethylolpropane triacrylate	7.5
1-Hydroxycyclohexyl phenyl ketone	0.375
Sanmorin OT-70[(a)]	0.259
Distilled water	9.0
Kayaset Blue N[(b)]	0.02

[(a)] Sanmorin OT-70: mixture of 70 wt% dioctyl sodium sulfosuccinate, 16 wt% propylene glycol, and 14 wt% water.

[(b)] Kayaset Blue N (C.I. Solvent Blue 35): oil-soluble blue dye.

Table S4. Composition of mixture of red fluorescent emulsion (components in Table S2) and blue emulsion (components in Table S3).

Components	Amount (g)
Red fluorescent emulsion (Table S2)	17.13
Blue emulsion (Table S3)	0.394

Table S5. Composition of coumarin-6-containing emulsion in the oil phase.

Components	Amount (g)
Trimethylolpropane triacrylate	7.5
1-Hydroxycyclohexyl phenyl ketone	0.375
Sanmorin OT-70[(a)]	0.259
Distilled water	9.0
Coumarin 6[(b)]	0.02

[(a)] Sanmorin OT-70: mixture of 70 wt% dioctyl sodium sulfosuccinate, 16 wt% propylene glycol, and 14 wt% water.

[(b)]Coumarin 6: 3-(2-Benzothiazolyl)-7-(diethylamino)coumarin, oil-soluble fluorescent dye.

Table S6. Composition of mixture of colorless emulsion (components in Table S1) and coumarin-6-containing emulsion (components in Table S5).

Components	Amount (g)
Colorless emulsion (Table S1)	17.13
Coumarin-6-containing emulsion (Table S5)	0.902

Table S7. Composition of mixture of colorless emulsion (components in Table S1) and blue emulsion (components in Table S3).

Components	Amount (g)
Colorless emulsion (Table S1)	17.13
Blue emulsion (Table S3)	4.28

Table S8. Composition of Omnirad-819-containing emulsion.

Components	Amount (g)
Trimethylolpropane triacrylate	7.5
Omnirad 819[(a)]	0.038
Sanmorin OT-70[(b)]	0.259
Distilled water	9.0

[(a)] Omnirad 819: bis(2,4,6-trimethylbenzoyl) phenylphosphine oxide (BAPO).

[(b)] Sanmorin OT-70: mixture of 70 wt% dioctyl sodium sulfosuccinate, 16 wt% propylene glycol, and 14 wt% water.

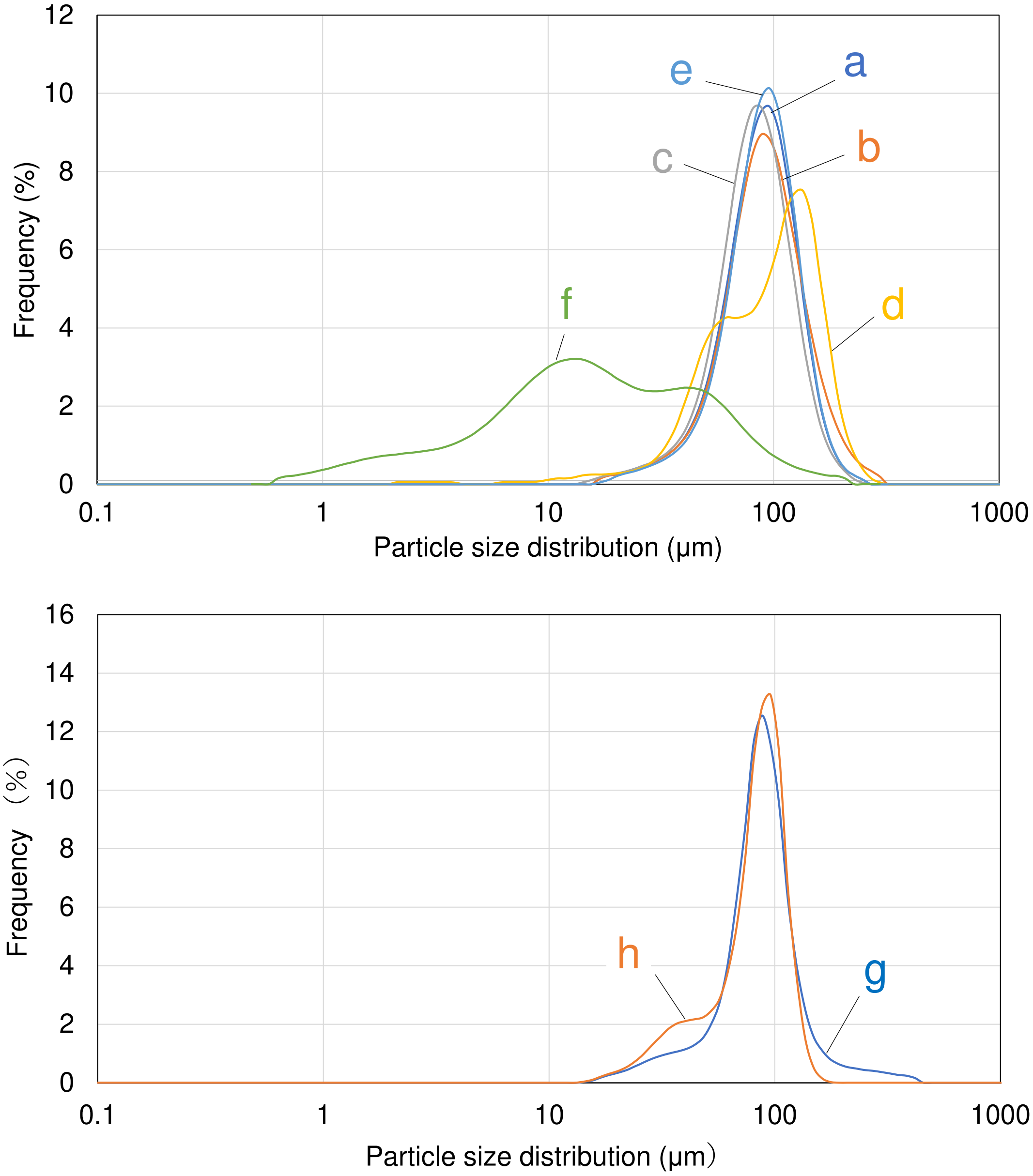

Figure S1. Particle size distribution of UV-curable crude emulsions employed in this study. The letters (a–e), (g), and (h) indicate droplet dispersions emulsified using a paint shaker, and (f) indicates that emulsified by intermittent manual shaking. (g) contains coumarin 6 (see components in Table S5). (h) contains Kayaset Blue N (see components in Table S3). The weight-average particle sizes obtained were (a) 87.7, (b) 92.9, (c) 82.8, (d) 96.8, (e) 89.3, (f) 14.9, (g) 88.9, and (h) 77.5 μm.

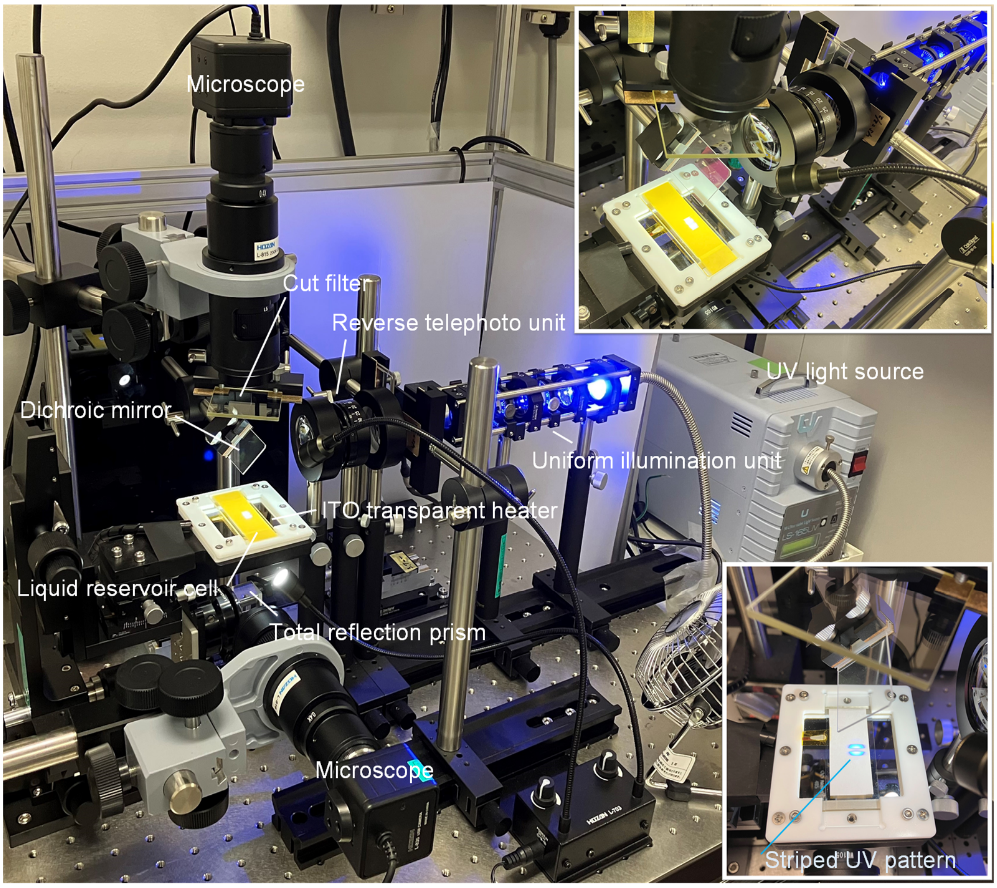

Figure S2. Photograph of the in situ microscopy observation optical system.

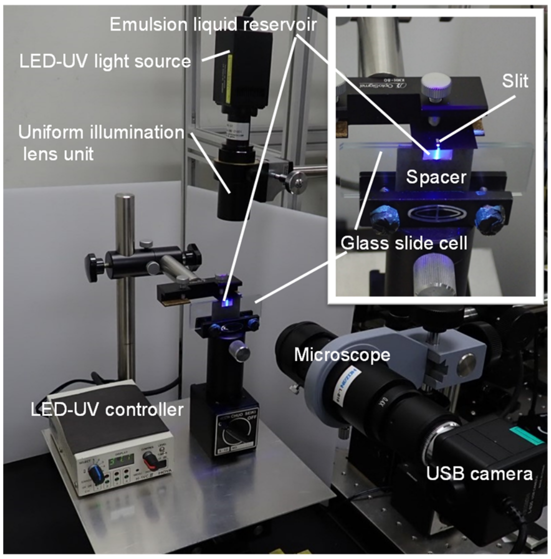

Figure S3. Photograph of the cross-sectional observation system for the emulsion liquid film.

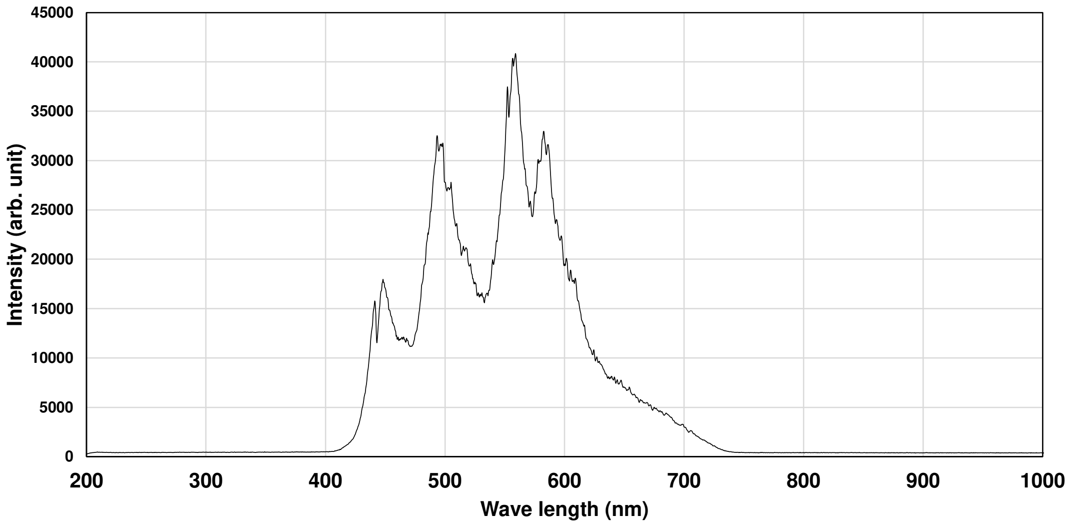

Figure S4. Spectrum of the projector used for the maskless exposure experiment.

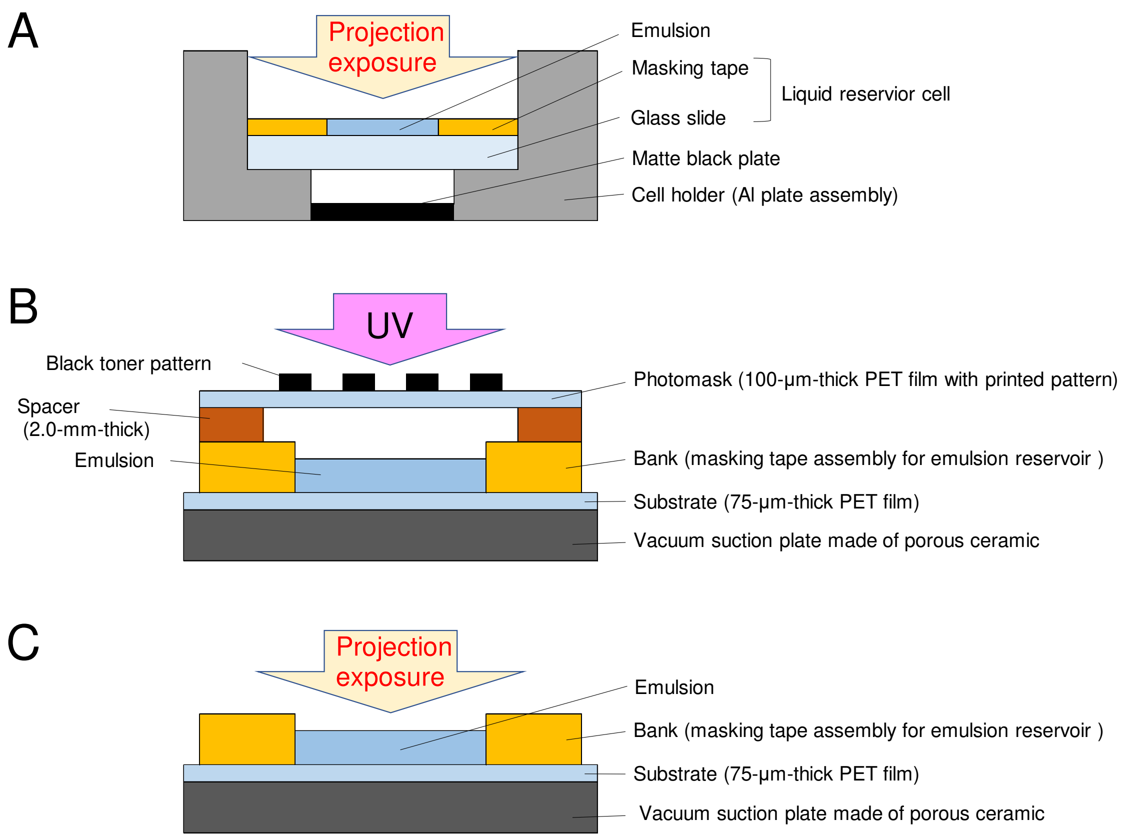

Figure S5. Layout of patterning experiments in ET method. (A) Projection exposure of the glass slide cell (liquid reservoir cell). (B) Pattern exposure of the emulsion filled onto PET film substrate through photomask. (C) Projection exposure of the emulsion filled onto PET film substrate.

Movie S1. In situ microscopy observation of the dynamic behavior of an emulsion in a liquid film under circular UV pattern exposure with reflected illumination. The diameter of the circular pattern was 3 mm. The UV illuminance was 11.25 mW/cm^2. Movie was taken using the observation system shown in Fig. 3A (see photo in Fig. S2). An LED white light source was used for illumination.

Movie S2. In situ microscopy observation of the dynamic behavior of an emulsion in a liquid film under circular UV pattern exposure with transmitted illumination. The UV circular pattern diameter, UV illuminance, observation system used for video recording, and lighting source were the same as those for Movie S1.

Movie S3. In situ microscopy observation of an emulsion under stripe UV pattern exposure for coumarin-6-containing emulsion blend system. The UV illuminance was 85.3 mW/cm^2 and the UV irradiation duration was 20 s. Immediately after the end of exposure, the film was dried at 80 °C using an ITO transparent heater. The heater reached 80 °C in about 20 s. The microscopy observation system for video recording was the same as that for Movie S1. Movie was taken from the back side of the liquid film through a total-reflection prism. The patterned film after drying was illuminated from the front side of the cell with a handy UV lamp (λ=365 nm), and the final positions of fluorescent coumarin-6-containing particles were recorded.

Movie S4. In situ microscopy observation of a blue emulsion blend system under stripe UV pattern exposure from the back side view of the liquid film. The observation system used for video recording was the same as that for Movie S1. The UV illuminance was 11.25 mW/cm^2 and the UV irradiation was for 20 s. Movie was taken from the back side of the liquid film through the total-reflection prism. Transmitted illumination was applied using an LED white light source from the front side of the cell.

Movie S5. In situ microscopy observation of a blue emulsion blend system under stripe UV pattern exposure from the front side view of the liquid film. The observation system and UV exposure conditions were the same as those for Movie S4. Movie was taken from the front side of the liquid film. Transmitted illumination was applied using an LED white light source from the back side of the cell.

Movie S6. Emulsion movement when exposed with a UV stripe pattern under overexposure condition and the formation of ET fog in unexposed areas with raised portion at the center. Microscopy movie was taken under reflected illumination. A UV stripe pattern with an illuminance of 113.6 mW/cm^2 was applied for about 20 s. Immediately after the end of exposure, the ITO transparent heater was turned on to start heating to 80 °C and turned off when the completion of drying was visually confirmed. The heater reached 80 °C in about 20 s after switching the power on.

Movie S7. Microscopy movie of the emulsion liquid film under UV partial irradiation taken from the film cross-sectional direction. The UV illuminance was 9.8 mW/cm^2, the width of the slit mask was 1 mm, and the distance between the slit and the surface of the emulsion liquid film was about 2.5 mm. Movie was taken using the observation system shown in Fig. 8A (see photo in Fig. S3).
